# 

**Published:** 1896-11-07

**Authors:** 


ABSOLUTELY PURE, therefore BEST,

**CADBURY'S COCOA**

"The standard of highest purity at present attainable."—*Lancet.*

**CADBURY'S COCOA**

NO ALKALIES USED.

### THE HOSPITAL

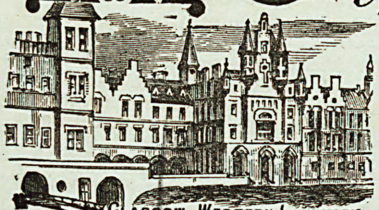

GLASGOW WESTERN INFIRMARY

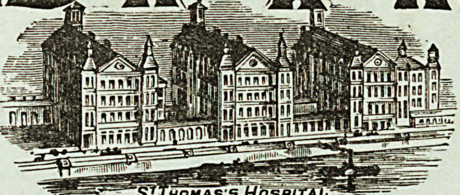

ST THOMAS'S HOSPITAL

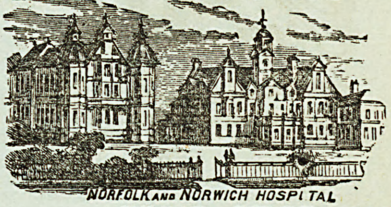

NORFOLK AND NORWICH HOSPITAL

No. 528. Vol. XXI.

SATURDAY, NOV. 7TH, 1896.

WITH EXTRA NURSING SUPPLEMENT.

[Registered at the General Post Office as a Newspaper for transmission in the United Kingdom and Abroad.]

PRICE TWOPENCE.

Per Annum, 10s 6d., post free.

Monthly Parts, 6d.; post free, 8d.

Ditto per Annum, 8s. 6d., post free.

